# The Need for Objective Measures of Stress in Autism

**DOI:** 10.3389/fpsyg.2017.00064

**Published:** 2017-01-27

**Authors:** Cédric Hufnagel, Patrick Chambres, Pierre R. Bertrand, Frédéric Dutheil

**Affiliations:** ^1^CNRS, LaPSCo, Stress Physiologique et Psychosocial, Université Clermont AuvergneClermont-Ferrand, France; ^2^CNRS, LMBP, Université Clermont AuvergneClermont-Ferrand, France; ^3^Preventive and Occupational Medicine, University Hospital of Clermont-Ferrand (CHU)Clermont-Ferrand, France; ^4^Faculty of Health, Australian Catholic UniversityMelbourne, VIC, Australia

**Keywords:** stress, anxiety, biomarker, autism, heart rate variability, objective measures

## Physiological and psychological impact of stressors

Despite the numerous definition of stress, the meaning of stress could refer to the adaptive behavioral or mental responses willing to address the common life consequences of stressors, such as increased attention to perform a mentally demanding task. The stressor can be real or perceived, pleasant, or unpleasant (Woda et al., 2016). Permanent adaptation to normal daily stressors is needed, via the physiological stress system. The physiological stress response triggers metabolic adaptations to the acute stressors (via activation of the autonomic nervous system mostly resulting in release of epinephrine by the adrenal medulla) and anticipates what may happen (via activation of the hypothalamo-pituitary adrenal axis resulting in release of corticosteroids by the cortical medulla) (McEwen, 2000; Woda et al., 2016). Morbid consequences can be expected when an individual is affected by a failure of the stress response system to stressors. Therefore, in its commonplace, the term “stress” is often viewed as a negative concept, with morbid consequences (Woda et al., 2016). One of these negative effects is anxiety. With anxiety, fear overcomes all emotions and is accompanied by worry and apprehension (Sylvers et al., 2011; Adhikari, 2014). While there is a definite overlap between stress and anxiety, we will use the term stress as a negative physiological and psychological impact of stressors.

## Stress in autism

People with autism spectrum disorder often have difficulties in communication and social interaction resulting from atypical information processing and abnormalities in sensory integration. This causes a cognitive and emotional overload state associated with an increased stress, by the involvement of the autonomic nervous system, that can lead to the appearance of inappropriate social behavior. However, in most actual publications (Reaven et al., 2012; Corbett et al., 2016; Bishop-Fitzpatrick et al., 2017), the stress of individuals with autism spectrum disorder is evaluated by questionnaires or sometimes by saliva biomarkers. Despite the lack of consistency between scores to questionnaires and levels of saliva biomarkers (Corbett et al., 2009; Spratt et al., 2012), they are not a direct and continuous assessment. This presupposes that caregivers or people with autism are able to recognize external and internal symptoms of stress, but also that stress systematically triggers an identifiable or observable behavioral response. We believe that stress evaluation should not be subjective. Individuals with autism spectrum disorder should benefit from objective continuous measures of stress, especially knowing that almost half of individuals with autism do not have access to effective communication to express this inner stress (American Psychiatric Association, 2013).

## Biomarkers of stress

Today most of stress assessment is done with saliva biomarkers such as cortisol or dehydroepiandrosterone (DHEA) levels, or its sulfated form (DHEA-S) (Danhof-Pont et al., 2011; Lac et al., 2012). However, those biomarkers do not give a direct and instantaneous assessment of stress or anxiety. They need to be transported in a cool storage for assessment in a dedicated laboratory. The levels of those biomarkers reflect a level of stress which may vary from some minutes ago to several hours ago, depending on their half-life. For example cortisol has a short half-life of 20 min, and thus may reveal acute stress; whereas DHEA-S has a long half-life of 16 h and thus reveals the global stress of a long period (half a day) (Woda et al., 2016). Moreover, DHEA-S levels will need a long period of time (several half-life) to return to basal values. Therefore, DHEA-S is a biomarker of chronic stress. Besides, putative biomarkers of stress which need blood sample are excluded because of feasibility. Even if we do not consider the high cost of those biomarkers, they also lack of specificity and conflicting results are reported (Oliveira et al., 2013; Fancourt et al., 2015; Hawn et al., 2015; Qi et al., 2016).

## The need for a continuous monitoring of stress

Real-time detection of stress needs continuous monitoring. To assess stress in daily life, we also need portable device. These devices must be non-invasive and pain-free. For these reasons, all historical markers of stress measured in blood, saliva, or urine, are excluded. The need to adapt to external and internal events involve the activation of the autonomous nervous system, which is a balance between sympathetic and parasympathetic activity (Shaffer et al., 2014). Vagal tone is considered to be a measure of parasympathetic activity which controls the resting state of internal organs via the vagus nerve. The most precise measure of the vagal tone is provided via its effect on heart rate. The vagal control of heart induces an increased heart rate variability (HRV) (Park and Thayer, 2014; Scott and Weems, 2014). HRV is the variation between two consecutive beats: the higher the variation, the higher the parasympathetic activity. A high HRV reflects the fact that an individual can constantly adapt to micro-environmental changes. An overload of stress induces a decrease in HRV and the adaptation mechanisms are exceeded. Therefore, low HRV is both a marker of cardiovascular risk and a biomarker of stress (Dutheil et al., 2012; Boudet et al., 2017). Conveniently, the measurement of HRV is easy, non-intrusive and pain-free, and provides continuous monitoring of the activity of the autonomic nervous system.

## Measuring heart rate variability

The most accurate way of proceeding HRV is to use a Holter-electrocardiogram which is a small medical device applied on the chest. A Holter-electrocardiogram give the exact time in milliseconds between two consecutive beats, based on R waves (Malik et al., 1996). The Holter-electrocardiogram is expensive, need to be precisely placed, and can cause some discomfort to wear. Therefore, heart rate transmitter belts now propose reliable measure of HRV (Akintola et al., 2016; Hernando et al., 2016). Due to the important amount of data to be processed, HRV require offline analysis witch is not compatible with real-time evaluation of stress. New method in development, that use detection of abrupt changes in HRV, will allow the identification of stressful events (Azzaoui et al., 2014; Dutheil et al., 2015). Heart rate, and thus HRV, are one of the easiest physiological measurements accessible to the general public. A heart rate transmitter belt is a budget option with accurate measures, but not as practical as and still more obtrusive than a simple wrist-band. Some wrist-based sensors are available but still lack of resolution to be used.

## The detection of abrupt changes

**Figure 1:**
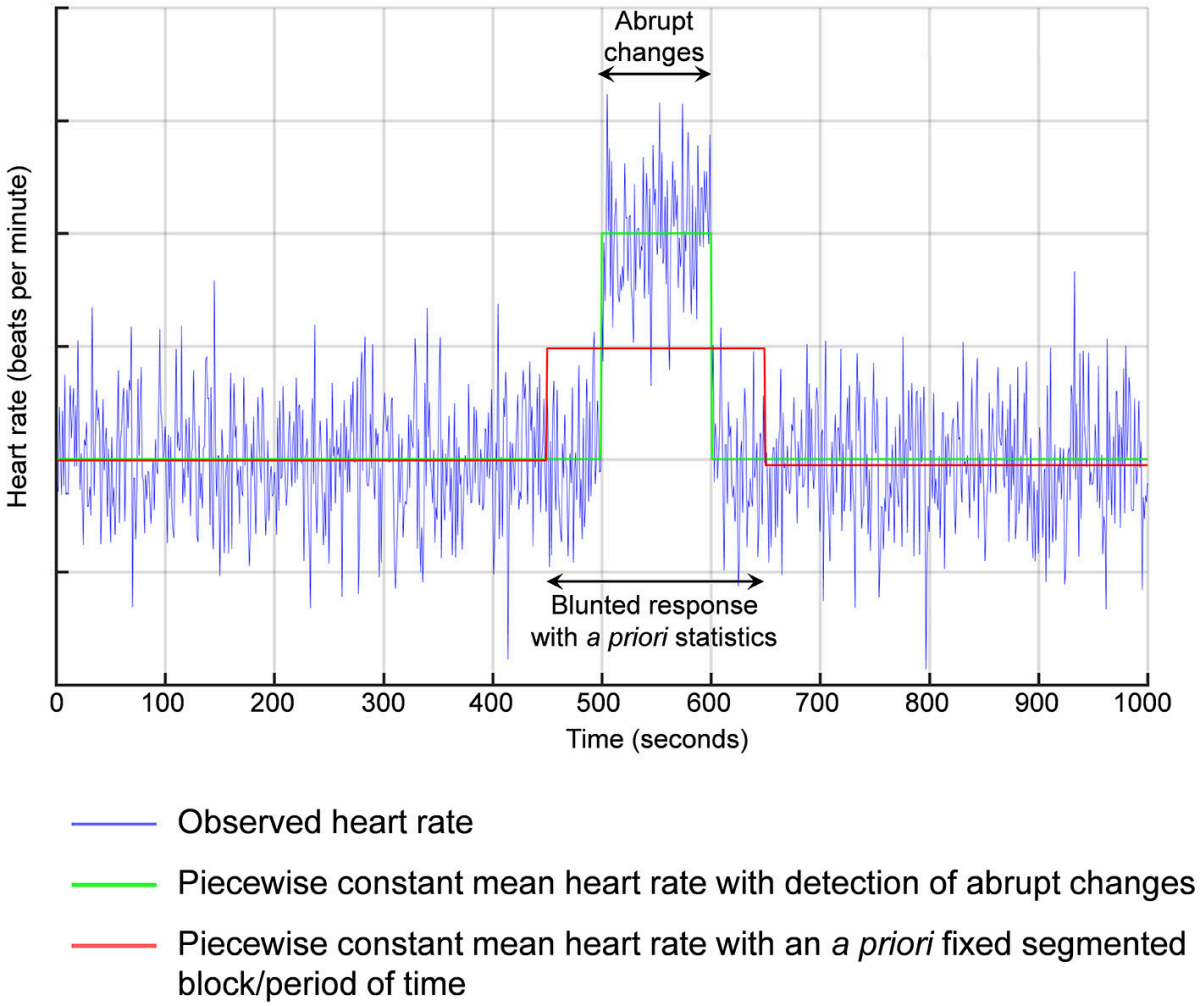
**Detection of abrupt changes**. For example, if the response in heart rate is delayed from the acute stress, previous methods using *a priori* segmented periods of time would average between the basal values and the stress reaction leading to a blunted response.

Previous literature have reported either normal or impaired baseline HRV in people with autism spectrum disorder (Cheshire, 2012; Kushki et al., 2014). Similarly, even if people with autism and healthy controls may share similar pattern of autonomic modifications following an acute mental stress (Kushki et al., 2014), some authors also reported a blunted HRV response to an acute stress (Hollocks et al., 2014, 2016). Despite the responses were still linked with stress, methods to analyze HRV response are questionable. Electrocardiogram recording are typically segmented into *a priori* blocks of 5 min each or other *a priori* fixed period of time. Our change point method is statistically different. We detect the abrupt change, then we calculate the mean value of HRV between two consecutive abrupt changes (Figure 1). The detection of abrupt changes is a statistical approach based on an individual basis and not on a population normalized level. There is no need for a control group. Personalized statistics are computed within the time-series of each individual, precluding bias. Detection of abrupt changes has a short history in medicine but a long history in quantitative finance, which has led to several Nobel prizes (Mandelbrot, 1963; Engle and Granger, 1982; Hansen, 1982; Granger, 2004).

## Stress assessment in daily life

However, most studies which evaluated HRV on individuals with autism were in laboratory and not in real daily life. Being in a laboratory is a challenging task by itself for people with autism spectrum disorder, and can generate hyper-reactivity of the autonomic nervous system (van Steensel et al., 2011; Jurko et al., 2016). Recent development in device portability, as well as data processing, give the opportunity to conceive new devices that can be used in daily life to perform stress assessment (El Kaliouby et al., 2006; Picard, 2009). Soon, just a watch should provide reliable continuous HRV monitoring. These watches could easily be connected to a smartphone application designed for the online detection of abrupt changes. These innovative data processing solutions will allow a live insight of the stress levels to be provided to individuals with autism and to their caregivers. Ultimately, this knowledge should allow appropriate intervention, particularly through the teaching of self-responses in different social contexts, thus limiting the emergence of disruptive behaviors (Dawson, 2008). Possibilities are not limited to autism spectrum disorders and many conditions or working situations should benefit from objective measures of stress.

## Author contributions

CH, Drafting the article. PC and PRB, Critical revision of the article. FD, Drafting the article, Final approval of the version to be published.

### Conflict of interest statement

The authors declare that the research was conducted in the absence of any commercial or financial relationships that could be construed as a potential conflict of interest.
